# Socio-economic inequality in the prevalence of violence against older adults – findings from India

**DOI:** 10.1186/s12877-021-02234-6

**Published:** 2021-05-20

**Authors:** Debashree Sinha, Prem Shankar Mishra, Shobhit Srivastava, Pradeep Kumar

**Affiliations:** 1 Department of Development Studies, International Institute for Population Sciences, Mumbai, Maharashtra 400088 India; 2 Population Research Centre, Institute for Social and Economic Change, Bengaluru, Karnataka 560072 India; 3 Department of Mathematical Demography & Statistics, International Institute for Population Sciences, Mumbai, Maharashtra 400088 India

**Keywords:** Violence, Older adults, Socio-economic inequality, India

## Abstract

**Background:**

Violence against older adults is a well-recognised socio-psychological and public health problem. It is uncared-for, undiagnosed, and an untreated problem that is widespread across both developed and developing countries. The present paper aims to understand the extent of the socio-economic status related inequality in violence against older adults in India.

**Methods:**

The study uses data from Building a Knowledge Base on Population Aging in India (BKPAI). Violence against older adults is the outcome variable for the study and is defined as *older adults who faced any abuse or violence or neglect or disrespect by any person.* Bivariate analysis and regression-based decomposition technique is used to understand the relative contribution of various socio-economic factors to violence against older adults (*N* = 9541).

**Results:**

The prevalence of violence faced by older adults was 11.2%. Older adults aged 80+ years [OR: 1.49; CI: 1.14–1.93] and working [OR: 1.26; CI: 1.02–1.56] had higher likelihood to face violence than their counterparts. On the other hand, older adults who were currently in union [OR: 0.79; CI: 0.65–0.95], lived with children [OR: 0.53; CI: 0.40–0.72] and who belonged to richer wealth quintile [OR: 0.35; CI:0.24–0.51] had lower likelihood to suffer from violence than their counterparts. The decomposition results revealed that poor older adults were more prone to violence (Concentration index: − 0.20). Household’s wealth status was responsible for explaining 93.7% of the socio-economic status related inequality whereas living arrangement of older adults explained 13.7% of the socio-economic related inequality. Education and working status of older adults made a substantial contribution to the inequalities in reported violence, explaining 3.7% and 3.3% of the total inequality, respectively.

**Conclusion:**

Though interpretation of the results requires a cautious understanding of the data used, the present study highlights some of the relevant issues faced by the country’s older adults. With no or meagre income of their own, older adults belonging to the poorest wealth quintile have little or no bargaining power to secure a violent free environment for themselves. Therefore, special attention in terms of social and economic support should be given to the economically vulnerable older population.

## Background

Globally in 2015, 900 million people were aged 60 years and above and it is speculated that there will be an increase of 2 billion older adults in 2050 [1]. Similarly. the population belonging to WHO’s South-East Asia region is also ageing rapidly; for instance, the proportion of people aged 60 or above was 9.8% in 2017, and it is expected to increase to 13.7 and 20.3% by 2030 and by 2050, respectively [2]. India is no exception to the phenomenon of ageing. For example, according to the 2011 Census, India contributes 8.6% of the aged population and further, it is expected to increase to 20% in 2050 [3]. The increase in the aged population in every corner of the world has led to social and health problems and is accompanied by various forms of violence peretrated against the older adults.

The World Health Organization defines violence against older adults or elderly abuse as “a single or repeated act or lack of appropriate action, occurring within any relationship where there is an expectation of trust which causes harm or distress to an older person” [4]. In most of the cases older adults are abused by their own family members, spouses, friends, community members, and also by healthcare providers. There are multiple forms of violence and abuse that have been reported. These include physical, sexual, psychological, and emotional abuse; financial and material abuse; abandonment; neglect; and serious loss of dignity and respect [4–6].

Violence against older adults is a socio-psychological and public health problem that is well recognised across the world. It is largely undiagnosed, uncared-for, and an untreated problem that is widespread across both developed and developing countries [7–10]. It is reported that around one in six people aged 60 years and above experience some form of abuse or violence in the community or household level settings [4]. This has eventually led to devastating consequences like serious injuries, health, and long-term socio-economic and psychological problems experienced by the older adults [9, 11–14]. Moreover, abuse and violence against older adults is one of the most serious socio-psychological-health problem in low and middle-income countries [15], which is growing at an unprecedented rate; and can be seen in South Asian countries like India [16, 17]. The lack of appropriate approach, neglect, and underreport of violence by older adults in these settings make it even more challenging. Further, violence is inversely associated with older adults’ quality of life, and directly associated with morbidity and mortality rates [5, 13, 18–20]. The experience of abuse or any kind of violence faced by the older adults is also linked to their disability and functional limitation [18]. Again, several socio-economic determinants influence higher risk of violence faced by older adults’ such as if the older person has low education, belongs to low-income group, and poor social status [3, 9, 12]. Studies have found that there is a relationship between caste and violence [18, 21], education and violence [12] low-income support and violence [22], unemployment and violence [23], poverty and violence against older adults [23]. Although there are other facets of certain risk factors that create an environment against older adults in physical and verbal abuse, however, it has not been extensively understood in the Indian scenario. Therefore, it becomes important to understand it thoroughly in the Indian context.

Though most of the studies found that a high incidence of violence against older adults exists in low socio-economic strata of the society, but it has not been frequently reported in many communities and therefore is highly underreported [3, 9, 12, 24]. Nevertheless, sometimes older adults feel the act of omission and commission in terms of violence or abuse, or mistreatment and they rarely report it. Until and unless it is an act of physical and verbal abuse or violence, they might not report it. The low coverage of such reporting is caused by several reasons such as inaccessible institutional support and lack of information, education, and communication [3, 12]. For understanding this, a systematic review presented and identified the major causes like the older adult faces nearly 11.6% psychological abuse, 6.8% financial abuse, 4.2 neglect, 2.6% physical violence, and 0.9% sexual abuse [15]. Similarly, a study in India showed that nearly 11% of older adults have experienced some form of violence after turning the age of 60 [12]. However, this significantly varies across sex and income groups. Existing literature emphasized the trends and patterns of older adults’ violence in India that are consistently linked to their health and socio-psychological well-being [9, 11, 25, 26].

## Methods

### Data

The present study utilized data from the Building a Knowledge Base on Population Aging in India (BKPAI) which was a national level survey and was conducted in 2011, across seven states of India [27]. The study was cross-sectional in nature. The survey was sponsored by Tata Institute for social sciences (TISS), Institute for social and economic change (ISEC), Institute for economic growth (IEG) and UNFPA, New Delhi [27]. The survey gathered information on various socio-economic, demographic and health aspects of ageing among households with members aged 60 years and above [27]. Seven major regionally representative states were selected for the survey with the highest 60+ year’s population than the national average [27]. This survey was carried out on a representative sample in the northern, western, eastern, and southern parts of India following a random sampling process [27]. The questionnaires for each state were bilingual, with questions in both the primary language of the states and English [27].

The primary sampling unit (PSU) were villages for rural areas, and urban wards in urban areas [27]. The sample of 1280 elderly households was fixed for each state. Further details on the sampling procedure, the sample size is available in national and state reports of BKPAI, 2011 [27]. The actual sample size was 9852 older adults aged 60 years and above. For the current study, after removing the missing cases (311 cases) the effective sample size was 9541 older adults residing in seven states aged 60+ years were selected [27].

#### Outcome variables

The outcome variable was binary in nature. The question was asked to older adult that *“Ever since you completed 60 years of age, have you faced any abuse or violence or neglect or disrespect by any person?”* The response was coded as 0 “No” and 1 “Yes”. Type of violence included were physical abuse, verbal abuse, economic abuse, showing disrespect and neglect [17].

#### Predictor variables

The predictor variables were included after doing extensive literature review:

1. Age in years (60–69, 70–79 and 80+) [28, 29].
2. Sex (men and women) [28, 29].
3. Education (none, below 5 years, 6–10 years and 11+ years) [28, 29].
4. Marital status (not in union and currently in union) [28–30].
5. Living arrangement (alone, with spouse, with children and others “includes other family members/relatives”) [29].
6. Working status (no and yes) [28–31].
7. Contributed money to household expenditure (no income, yes and no) [29].
8. Wealth (poorest, poorer, middle, richer and richest) [28–33]. The wealth index was based on the following 30 assets and housing characteristics: household electrification; drinking water source; house ownership; type of toilet facility; type of house; ownership of a bank or post-office account; cooking fuel; and ownership of a mattress, a pressure cooker, a cot/bed, a table, a chair, an electric fan, a radio/transistor, a black and white television, a colour television, a mobile telephone, any landline phone, a sewing machine, a computer, internet facility; a refrigerator, a watch or clock, a bicycle, a motorcycle or scooter, an animal-drawn cart, a car, a water pump, a thresher and a tractor [28–31]. The range of index was from poorest to the richest i.e. ranging from lowest to the highest [28–34].
9. Religion (Hindu, Muslim, Sikh and others) [28–31].
10. Caste (Scheduled Caste (SC), Scheduled Tribe (ST), Other Backward Class, and Others) [28–32].
11. Place of residence (rural and urban) [28–31].
12. States (Himachal Pradesh, Punjab, West Bengal, Odisha, Maharashtra, Kerala and Tamil Nadu) [28–31].

### Research framework

There are many theories such as social exchange theory, feminist theory, political-economic theory, psychopathology of the caregiver theory, role accumulation theory, situational theory, social learning theory, stratification theory, and symbolic interactionism theory that provides probable causes of older adults facing abuse and violence [35]. Similarly, other studies have proposed interventions to prevent elder abuse by lessons learned from child abuse and intimate partner violence [36]. However, after an extensive review of literature on violence faced by older adults, we have tried to develop a research framework that highlights the important role of socio-economic characteristics as risk factors to violence experienced by older adults in India.

**Fig. 1:**
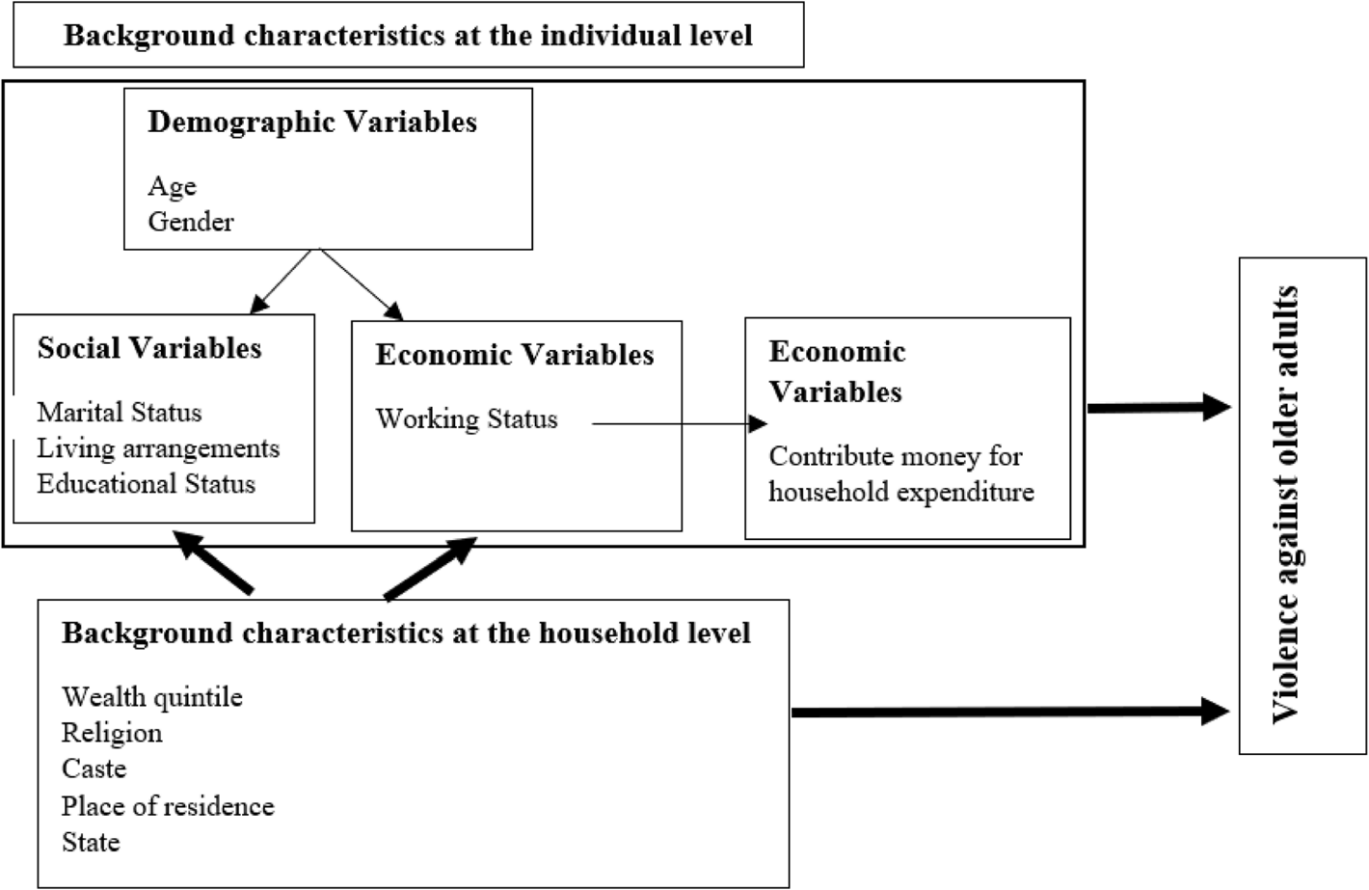
Research framework: risk factors to violence experiences by older adults

In the above Fig. 1, an older adult’s household background characteristics like wealth quintile, religion, caste, place of residence, and state influence his individual, social and economic characteristics. Again, within the individual-level characteristics, an older adult’s age and sex influence his/her social and economic characteristics. Further, an older adult’s working status influences how much he can contribute to household expenditure. Finally, both individual and household level characteristics of an older adult affect his exposure to violence. The research framework also shows the link between various independent variables and the dependent variable. Thus, based on the above research framework and existing literature, this paper aims to understand the extent of socio-economic status related inequality in violence against older adults in the Indian society. The The effort is made to identify the most vulnerable population sub-group who suffers from different types of violence.

#### Statistical analysis

Descriptive statistics along with bivariate analysis were used to find the preliminary results [29]. Further, binary logistic regression analysis [37] has been done to fulfil the objective of the study. The results were presented in the form of adjusted odds ratio (OR) with a 95% confidence interval (CI) [28].

The model is usually put into a more compact form as follows:

$$ \ln \left(\frac{P_i}{1-{P}_i}\right)={\beta}_0+{\beta}_1{x}_1+\dots +{\beta}_M{x}_{m-1}, $$

Where *β*_0_, …. . , *β*_*M*_ are regression coefficient indicating the relative effect of a particular explanatory variable on the outcome [29].

Moreover, wealth quintile was used as the key variable to measure the socio economic status of the particular household household [38]. A household wealth index was calculated in the survey by combining household amenities, assets and durables and characterizing households in a range varying from the poorest to the richest, corresponding to wealth quintiles ranging from the lowest to the highest [28].

The study used continuous wealth score for decomposition analysis and for the calculation of Concentration Index (CCI) [30, 31]. The study used wealth quintile which has been divided into five equal size of the population [30, 31].

#### Concentration index

Concentration index reveals the magnitude of inequality by estimating the area between the concentration curve and line of equality [30, 31], and calculated as two times the weighted covariance between the explanatory and fractional rank in the wealth distribution divided by variable mean [33].

The concentration index can be written as follows:

$$ \boldsymbol{C}=\frac{\mathbf{2}}{\boldsymbol{\mu}}\boldsymbol{\operatorname{cov}}\left({\boldsymbol{V}}_{\boldsymbol{i},}{\boldsymbol{W}}_{\boldsymbol{i}}\right) $$

Where, C is the concentration index; *V*_*i*_ is the outcome variable index; ***W*** is the fractional rank of individual ***i*** in the distribution of socio-economic position; ***μ*** is the mean of the outcome variable of the sample and ***cov*** denotes the covariance [39]. The index value lies between − 1 to + 1 [30, 31].

Further, the study decomposed the concentration index to understand the relative contribution of various socioeconomic factors to violence faced by older adults [40]. To do this, the study used regression based decomposition technique, which was proposed by Wagstaff et al. [41].

## Results

Socio-demographic profile of older adults and percentage of older adults who faced any violence by background characteristicsis are presented in Table 1. About three-fifth of older adults belonged to 60–69 years age group and nearly half of them were women. Only 6 % of older adults had more than 11 years of education and same proportion of older adults were living alone. Around one-fourth of older adults were working and half of the older adults contributed money for household expenditure. Majority of older adults were Hindu and lived in rural areas. Further results show that older adults with 80+ years of age (15.4%), women (11.5%) and those who had no education (13.6%) reported more violence compared to their counterparts. Older adults with higher education and currently in union faced less any violence in the household. Older adults who lived alone (17.6%) and working (15.2%) reported more violence than those who lived with others and not working respectively. Older adults those who did not contribute money for household expenditure (15.4%) faced more any violence compared to therest of the categories. There was a negative association between wealth quintile and reporting of violence by older adults. A higher proportion of older adults belonged to Scheduled Tribe and lived in rural areas reported more violence than other caste categories and those who lived in urban areas. The highest percentage of violence was reported in Maharashtra (34.3%) followed by Himachal Pradesh (11.5%).

**Table 1:** Socio-economic profile of older adults and percentage of older adults who faced any violence by background characteristics in India

Variables	Sample	Percentage	Any violence (%)	***p***-value
**Age (years)**				0.008
60–69	5890	61.8	10.7	
70–79	2612	27.4	10.7	
80+	1036	10.9	15.4	
**Sex**				0.029
Men	4525	47.4	10.8	
Women	5014	52.6	11.5	
**Educational status**				0.001
None	4871	51.1	13.6	
Below 5 years	1954	20.5	11.8	
6 to 10 years	2136	22.4	6.6	
11+ years	578	6.1	6.0	
**Marital status**				0.001
Not in union	3759	39.4	12.3	
Currently in union	5780	60.6	10.5	
**Living arrangement**				0.001
Alone	561	5.9	17.6	
With spouse	1521	15.9	12.2	
With children	6717	70.4	10.6	
Others	740	7.8	9.6	
**Working status**				0.001
No	7229	75.8	9.9	
Yes	2310	24.2	15.2	
**Contribute money for household expenditure**				0.034
No income	4110	43.1	10.7	
Yes	5013	52.6	11.2	
No	416	4.4	15.4	
**Wealth quintile**				0.001
Poorest	2251	23.6	17.3	
Poorer	2114	22.2	13.1	
Middle	1970	20.7	8.4	
Richer	1771	18.6	8.0	
Richest	1433	15.0	6.4	
**Religion**				0.187
Hindu	7570	79.4	11.1	
Muslim	671	7.0	10.0	
Sikh	898	9.4	12.4	
Others	400	4.2	12.9	
**Caste**				0.045
Scheduled Caste	1979	20.7	12.0	
Scheduled Tribe	531	5.6	14.0	
Other Backward Class	3507	36.8	7.9	
Others	3522	36.9	13.5	
**Place of residence**				0.001
Rural	7042	73.8	12.2	
Urban	2497	26.2	8.3	
**State**				0.001
Himachal Pradesh	1470	15.4	11.5	
Punjab	1351	14.2	10.4	
West Bengal	1127	11.8	7.5	
Orissa	1453	15.2	9.2	
Maharashtra	1380	14.5	34.3	
Kerala	1356	14.2	2.9	
Tamil Nadu	1403	14.7	1.9	
**Total**	9539	100	11.2	

Results from logistic regression estimates for violence among older adults were presented in Table 2. The likelihood of violence was significantly higher among older adults with age 80+ years (OR, 1.49; CI: 1.14–1.93) compared to 60–69 years age group. Moreover, older adults currently in union (OR, 0.79; CI: 0.65–0.95) were less likely to face violence than those who were not in union. Older adults those who were working (OR, 1.26; CI: 1.02–1.56) reported significantly higher odds of violence compared to those who were not working. The likelihood of violence was higher among older adults those who did not contribute money for household expenditure (OR, 1.25; CI: 1.05–1.86) than reference category. The odds of violence was higher in Maharashtra (OR, 3.43 CI: 2.62–4.75) however it was lower in West Bengal (OR, 0.50; CI: 0.34–0.72), Orissa (OR, 0.56; CI: 0.39–0.82), Kerala (OR, 0.28; CI: 0.18–0.44) and Tamil Nadu (OR, 0.13; CI: 0.08–0.22) compared to Himachal Pradesh.

**Table 2:** Logistic regression estimates for violence among older adults by background characteristics in India

Background characteristics	Odds Ratio (95% CI)
**Age (years)**	
60–69	Ref.
70–79	0.97(0.78,1.19)
80+	1.49*(1.14,1.93)
**Sex**	
Men	Ref.
Women	0.96(0.78,1.19)
**Educational status**	
None	Ref.
Below 5 years	1.08(0.89,1.32)
6 to 10 years	0.79*(0.63,1)
11+ years	0.60*(0.39,0.9)
**Marital status**	
Not in union	Ref.
Currently in union	0.79*(0.65,0.95)
**Living arrangement**	
Alone	Ref.
With spouse	0.69*(0.48,0.98)
With children	0.53*(0.4,0.72)
Others	0.44*(0.3,0.66)
**Working status**	
No	Ref.
Yes	1.26*(1.02,1.56)
**Contribute money for household expenditure**	
No income	Ref.
Yes	0.92(0.74,1.16)
No	1.25*(1.05,1.86)
**Wealth quintile**	
Poorest	Ref.
Poorer	0.63*(0.5,0.79)
Middle	0.43*(0.32,0.58)
Richer	0.39*(0.29,0.53)
Richest	0.35*(0.24,0.51)
**Religion**	
Hindu	Ref.
Muslim	1.08(0.75,1.55)
Sikh	1.51*(1.04,2.18)
Others	1.09(0.72,1.66)
**Caste**	
Scheduled Caste	Ref.
Scheduled Tribe	0.91(0.63,1.32)
Other Backward Class	0.96(0.73,1.25)
Others	1.37*(1.07,1.75)
**Place of residence**	
Rural	Ref.
Urban	0.94(0.78,1.14)
**State**	
Himachal Pradesh	Ref.
Punjab	0.82(0.56,1.2)
West Bengal	0.50*(0.34,0.72)
Orissa	0.56*(0.39,0.82)
Maharashtra	3.53*(2.62,4.75)
Kerala	0.28*(0.18,0.44)
Tamil Nadu	0.13*(0.08,0.22)

*p < 0.05; *CI* Confidence Interval, *Ref* Reference category

**Fig. 2:**
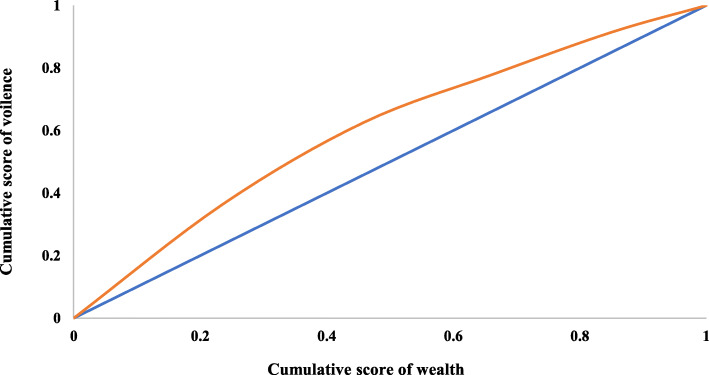
Concentration curve for violence among older adults in India

Figure 2 depicts the concentration curve for violence reported by older adults in India. Since the concentration curve lies above the line of equality it implies that violence among older adults is concentrated among the poor. If the curve was formed below the line of equality then the inequality would concentrate towards rich and vice-versa. Moreover, more the area between line of equality and curve higher the inequality. India was having inequality of − 0.20 which depicts that violence was concentrated more among poor older adults.

Estimates of decomposition analysis for the contribution of various explanatory variables to violence among older adults are presented in Table 3. The positive scores of concentration index denotes that violence among older adults concentrated among rich older adults for that particular predictor and vice-versa. Older adults aged 70–79 years, women, living with spouse, working, those contributing money for household expenditure, belonging poorer wealth quintile, and to SC/ST categories and living in urban areas concentrated more among disadvantaged population in terms of reported violence. On the other hand, having secondary or higher education, currently in union, living with children, and belonging to Muslim or Sikh religion inclined to concentrate among the better off. Household’s wealth status, living arrangement, education, and working status of older adults were the major contributors to the inequalities. Household’s wealth status was responsible for explaining 93.7% of the SES-related inequality whereas living arrangement of older adults explained 13.7% SES-related inequality. Education and working status of older adults made a substantial contribution to the inequalities in reported violence, explaining 3.7 and 3.3% of the total inequality, respectively.

**Table 3:** Estimates of decomposition analysis for contribution of various explanatory variables for violence among older adults in India

Background characteristics	Coefficient	Elasticity	CCI	Absolute contribution	% Contribution	
**Age (years)**						
60–69						
70–79	0.06	−0.001	−0.014	0.000	− 0.1	
80+	0.51*	0.004	0.018	0.000	− 0.3	− 0.4
**Sex**						
Men						
Women	−0.05	− 0.001	− 0.033	0.000	− 0.2	− 0.2
**Educational status**						
None						
Below 5 years	0.08	0.003	0.002	0.000	0.0	
6 to 10 years	− 0.23*	− 0.003	0.260	− 0.001	3.8	
11+ years	−0.52	0.000	0.613	0.000	0.0	3.7
**Marital status**						
Not in union						
Currently in union	−0.24*	−0.009	0.040	0.000	1.7	1.7
**Living arrangement**						
Alone						
With spouse	−0.37*	−0.005	− 0.197	0.001	−4.7	
With children	−0.63*	−0.038	0.089	−0.003	16.3	
Others	−0.81*	−0.005	0.092	0.000	2.2	13.7
**Working status**						
No						
Yes	0.23*	0.004	−0.174	−0.001	3.3	3.3
**Contribute money for household expenditure**						
No income						
Yes	−0.12	−0.003	−0.006	0.000	−0.1	
No	0.16*	0.001	−0.054	0.000	0.3	0.2
**Wealth quintile**						
Poorest						
Poorer	−0.38*	−0.012	− 0.338	0.004	−19.5	
Middle	−0.73*	−0.018	0.139	−0.003	12.0	
Richer	−0.91*	−0.017	0.523	−0.009	42.7	
Richest	−1.03*	−0.016	0.760	−0.012	58.5	93.7
**Religion**						
Hindu						
Muslim	0.03	0.001	0.146	0.000	−0.7	
Sikh	0.37*	0.004	0.311	0.001	−6.0	
Others	0.06	0.000	0.296	0.000	0.0	−6.7
**Caste**						
Scheduled Caste						
Scheduled Tribe	0.01	−0.001	−0.444	0.000	−2.1	
Other Backward Class	0.03	−0.001	−0.029	0.000	−0.1	
Others	0.30*	0.012	0.219	0.003	−12.6	−14.9
**Place of residence**						
Rural						
Urban	−0.07	−0.001	0.247	0.000	1.2	1.2
**State**						
Himachal Pradesh						
Punjab	−0.05	−0.003	0.331	−0.001	4.8	
West Bengal	−0.50*	−0.007	− 0.163	0.001	−5.5	
Orissa	−0.59*	−0.008	− 0.368	0.003	−14.2	
Maharashtra	1.29*	0.031	−0.125	−0.004	18.6	
Kerala	−1.06*	−0.010	0.349	−0.003	16.8	
Tamil Nadu	−2.04*	−0.015	−0.222	0.003	−16.0	4.5
**Total**					100.0	100.0
**Calculated CI**				−0.021		
**Actual CI**				−0.198		
**Residual**				−0.177		

*CCI* Concentration index; *if *p* < 0.05; %: percentage

## Discussion

Using the BKPAI data of 2011, the present paper tried to understand the socio-economic inequality for violence among 9541 older adults residing in the Indian states of Himachal Pradesh, Punjab, West Bengal, Odisha, Maharashtra, Kerala and Tamil Nadu. The key highlights of the present paper are as follows: First, the prevalence of violence faced by older adults is 11.2%. However, there is a considerable state-wise variation in the prevalence of violence. For example, while older adults in Tamil Nadu experience the least violence (1.9%), older adults in Maharashtra face the most (34.3%). Second, while the positive significant determinants of experiencing violence among the older adults are age and work status, the negative significant determinants are educational and marital status, living arrangement, and wealth quintile. Third, there exist clear evidence of socio-economic inequality in experiencing violence and the household’s wealth status contributed to the maximum; consequently, older adults belonging to the poorer sections of the society are more susceptible to violence. The results are in consistent with the proposed research framework where different socio-economic risk factors influence an older adult’s exposure to violence.

Elder abuse is prevalent worldwide but given the rapid ageing of population in Asia, the number of abused elders in Asia are also expected to rise [42]. For instance, a study in Nepal showed that the prevalence of elder abuse was 50.3% [43]. Again, in Bangladesh 27.2% of older adults faced elder abuse [44]. The situation in India is even more alarming. One-eighth of the world’s older adult lives in India. With falling income and health, breaking of the joint family and change in social attitudes, the older adults are the most vulnerable sub-population of the country. Coupled with this, abuse faced by older adult’s is becoming more and more prevalent in India [3, 17, 45]. The study results indicated that the overall prevalence of any violence faced by older adults is 11.2%. Similar studies that have investigated the prevalence of elder abuse in the Indian context by using the BKPAI data reports that 10–11% of older adults face abuse [12, 46]. Further, among the seven states included in the current data older adults in Maharashtra face the maximum abuse and the minimum is faced by older adults in Tamil Nadu. The finding is consistent with existing literature [46, 47]. Various other community based cross-sectional studies on elder abuse in India show that overall abuse rate can lie in the range from 9.36 to 25.6% [3, 48]. To better understand the situation of elder abuse in India, regular surveys are conducted by HelpAge India. According to the recent survey, it was reported that nearly 25% of older adults experience abuse in the country [49].

Our finding that the likelihood of experiencing violence increases with age contradicts with studies [50–52]. However, excerpts from focus group discussion on reasons of abuse among older adults due to age revealed that since the older adults are not young and do not belong to vibrant young culture, it is easy to being disrespected [53]. Education is found to be a significant negative predictor of experience of violence among older adults and is in line with other studies [11, 50, 51, 54, 55]. However, higher educational attainment may also affect the level of openness on abuse among the older adults and therefore, they might be unwilling to share information on sensitive topics like abuse to maintain a family façade [12]. A popular believe is that, those who are economically independent have lesser odds of experiencing violence [56]. Yet, our results indicated otherwise - older adults with working status experience higher odds of violence. The possible explanations for our study finding may be because the income earned is forcibly taken by household members suggesting the presence of economic violence. Further, those who do not contribute money for household expenses are more likely to experience violence because they are economically dependent on their children [57]. Therefore, our study results indicate that for older adults being employed (i.e., economically independent) as well as not contributing to household expenses (i.e., economically dependent), both act as risk factors to experience violence.

Our decomposition results indicate that there exists economic inequality in reporting of violence by older adults. A study by Naughton et al., 2012 in Ireland showed that individuals with a low-income had a doubled risk of being victims of abuse of any kind [58]. Likewise, low income was associated with neglect, but not when other forms of abuse was considered [59]. In Iran, Hosseinkhani, Zahra Khodamoradi & Sheikh, 2019 found that older adults belonging to lower socio-economic status were majorly at the risk of abuse [22]. The World Health Organization (WHO) and International Network for the Prevention of Elder Abuse (INPEA) in their study *Missing Voices* gathered the views of older persons on elder abuse and showed that poverty and inequality are reasons for elder abuse [60]. Keskinoglu et al., 2007 tried to explain the factors such as living with many family members and with low family income that cause violence towards older adults with low income [61]. Further, the rates of abuse among older adults are highest in families where income levels for the older adult and for the abuser/caregiver are extremely low [62] and, there is low coverage of social security for older adults that tends to increase the burden on their care givers [12].

Traditional Indian values have always perceived old age as a stage of wisdom, maturity, prestige, and power, with respect given to older adults, especially to the oldest male [23]. India also represents an orthodox and patriarchal society where older adults have been taken care of by their sons and daughters-in-law. Given the low coverage of social security for older adults in India, it tends to increase burden on families who support older adults [12]. A recent verbatim from focus group discussions reveal that older adults are now considered as burden in the society [45]. Further, of all the forms of violence, negligence, abandonment, and financial abuse are the most common form of violence faced by older adults in India [21, 26, 63]. However, most of the time these go underreported [24] since it is usually penetrated by family members in Indian society [24–26]. Additionally, along with physical disability, poor physical and mental health, the other associated risk factors of violence comprise of poor socio-economic condition of the older adults [3, 9, 64]. Literature found that older adults who belong to lower economic strata, lower caste group and having low education are more prone to abuse and violence compare to their counterpart [3, 12, 21, 57]. Therefore, violence faced by older adults has created social and health vulnerability [5, 20, 24].

Our study is not devoid of limitations. One of them is that the analysis is based on cross-sectional data that limited our scope to do cause-effect analysis. Second, one must be cautious while interpretating the results of the present study since the data used for analysis covers only the states of Himachal Pradesh, Punjab, West Bengal, Odisha, Maharashtra, Kerala and Tamil Nadu, and not all the states of the country. Third, the outcome variable did not measure the time reference like whether the respondent experienced violence 6 months back or a year back. Time reference is important to avoid recall bias or to get the accurate information. Fourth, since the question on elder abuse is sensitive in nature it depends on how the older adults have responded to it; various factors like whether the response was given in presence of others or not, whether he was stressed or not might affect the response given. Finally, since the data is a decade old so it cannot be generalized now.

## Conclusion

The results indicate that violence among older adults is prevalent in the Indian society and therefore, an understanding of its determinants is valuable for policy makers to improve the services towards the older adults of the country. The existence of economic inequality in reporting of violence shows the miserable conditions of the older adults belonging to the poorest wealth quintile. With no or meagre income of their own, they have little or no bargaining power to secure a violent free environment for themselves. Social and economic support should be given to these older adults to live a dignified life. Finally, keeping in mind, the changing pattern of Indian culture and values, efforts should be made to make individuals across all age group respect, honour and care for the older adults.

## Data Availability

We have provided details of the data in the methodology section. The BKPAI data can be obtained from the ISEC Bangalore. The report and the survey tools are also available on the website: https://india.unfpa.org/sites/default/files/pub-pdf/AgeingReport_2012_F.pdf

## Abbreviations

BKPAI: Building a Knowledge Base on Population Aging in India
ISEC: Institute for social and economic change
TISS: Tata Institute for social sciences
IEG: Institute for economic growth
PSU: Primary Sampling Unit
SC: Scheduled Caste
ST: Scheduled Tribe
OR: Odds Ratio
CCI: Concentration Index
SES: Socio Economic Status
WHO: World Health Organization
INPEA: International Network for the Prevention of Elder Abuse
